# Chemoradiotherapy enhanced the efficacy of radiotherapy in nasopharyngeal carcinoma patients: a network meta-analysis

**DOI:** 10.18632/oncotarget.16349

**Published:** 2017-03-17

**Authors:** Jian He, Ping Wu, Yaoyun Tang, Sulai Liu, Chubo Xie, Shi Luo, Junfeng Zeng, Jing Xu, Suping Zhao

**Affiliations:** ^1^ Department of Otorhinolaryngology Head & Neck Surgery, Province Key Laboratory of Otolaryngology Critical Diseases, Xiangya Hospital of Central South University, Changsha, Hunan, China

**Keywords:** nasopharyngeal carcinoma, chemoradiotherapy, overall survival, complete response, network meta-analysis

## Abstract

**Object:**

A Bayesian network meta-analysis (NMA) was conducted to estimate the overall survival (OS) and complete response (CR) performance in nasopharyngeal carcinoma (NPC) patients who have been given the treatment of radiotherapy, concurrent chemoradiotherapy (C), adjuvant chemotherapy (A), neoadjuvant chemotherapy (N), concurrent chemoradiotherapy with adjuvant chemotherapy (C+A), concurrent chemoradiotherapy with neoadjuvant chemotherapy (C+N) and neoadjuvant chemotherapy with adjuvant chemotherapy (N+A).

**Methods:**

Literature search was conducted in electronic databases. Hazard ratios (HRs) accompanied their 95% confidence intervals (95%CIs) or 95% credible intervals (95%CrIs) were applied to measure the relative survival benefit between two comparators. Meanwhile odd ratios (ORs) with their 95% CIs or CrIs were given to present CR data from individual studies.

**RESULTS:**

Totally 52 qualified studies with 10,081 patients were included in this NMA. In conventional meta-analysis (MA), patients with N+C exhibited an average increase of 9% in the 3-year OS in relation to those with C+A. As for the NMA results, five therapies were associated with a significantly reduced HR when compared with the control group when concerning 5-year OS. C, C+A and N+A also presented a decreased HR compared with A. There was continuity among 1-year, 3-year and 5-year OS status. Cluster analysis suggested that the three chemoradiotherapy appeared to be divided into the most compete group which is located in the upper right corner of the cluster plot.

**Conclusion:**

In view of survival rate and complete response, the NMA results revealed that C, C+A and C+N showed excellent efficacy. As a result, these 3 therapies were supposed to be considered as the first-line treatment according to this NMA.

## INTRODUCTION

Nasopharyngeal carcinoma (NPC), derived from the nasopharynx, is an epidemic cancer in Southeast Asian countries, Southeast China and North Africa [1]. NPC patients often were diagnosed at advanced stages and radiotherapy (RT) was used to be the recommended option for these patients [2]. However, only 30%-50% NPC patients with RT were able to survive for 5 years [3]. Meanwhile, chemical compounds like SSRP1 that are able to reduce the proliferation of NPC tumor cells was identified in previous studies [4]. As a result, the combination of chemotherapy and RT was hypothesized to be an effective therapy to improve the survival status of NPC patients. And such result was verified by studies in the current literatures [5].

Three primary chemoradiotherapies was introduced to control locoregionally advanced NPC: concurrent chemoradiotherapy, concurrent chemoradiotherapy plus adjuvant chemotherapy and concurrent chemoradiotherapy plus neoadjuvant chemoradiotherapy. It was revealed that the 3 mentioned methods worked in totally different mechanisms and focused on different purposes. For instance, chemoradiotherapy is prepared for the purpose of multiplying the treatment effects and neoadjuvant chemoradiotherapy is able to reduce the size of tumor before the implementation of RT. It was suggested that patients with neoadjuvant chemoradiotherapy exhibited a lower risk of recurrence in comparison to those with the monotherapy of RT [6].

Although some MA was conducted to compare different chemoradiotherapies, most randomized clinical trials (RCTs) can only compare two or three arms of therapies due to resource constraints and ethical issues. As a result, simultaneously comparison to the efficacy of several chemoradiotherapies cannot be achieved by RCTs or conventional meta-analysis. Therefore, the approach of mixed-treatment comparisons or network meta-analysis (NMA) was adopted in this study in order to overcome the above limitations. It was also expected to examine whether combined chemoradiotherapy was able to provide NPC patients with enhanced efficacy from this NMA. For this reason, evidence was synthesized from studies in which adjuvant chemotherapy, concurrent chemoradiotherapy, neoadjuvant chemotherapy or their binary combination therapies (A, C, N, A+C, N+C and A+N) were included and compared.. By conducting such a study, genuine consensus can be reached in the current literature which is critical to patients with NPC.

## RESULTS

### Baseline characteristics

**Figure 1:**
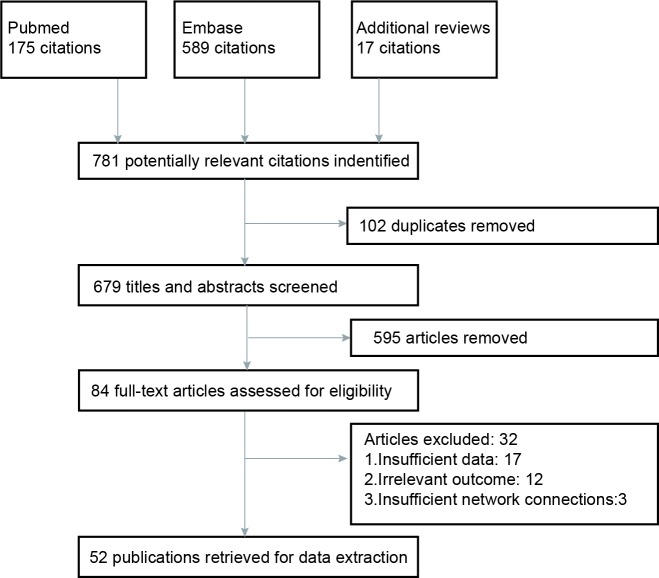
Flow chart of study selection

**Figure 2:**
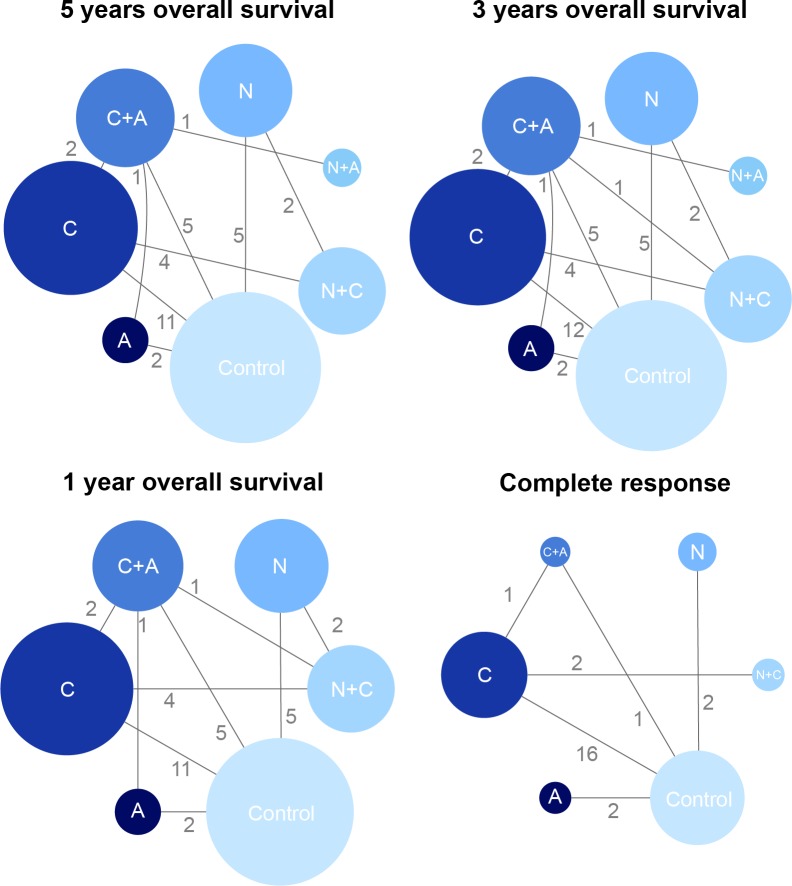
The network plot of included interventions

As was revealed in the flow chart (Figure 1), a total of 781 articles were identified by two reviewers (PubMed: 175, Embase: 598, additional reviews: 17). Then 595 irrelevant articles as accompanied with 102 duplicates were excluded, resulting in 84 articles for full-text assessment. After another 32 articles were removed, 52 publications with a total of 10,081 NPC patients were included in the eligibly study list. The baseline characteristics of included studies were shown in Table 1. Besides, the network plots revealing the distribution of trials for each outcome were shown in Figure 2. The size of nodes was proportional to the number of patients with that comparator and the numbers on the edges between two nodes indicated the number of included direct evidences.

**Table 1:** Characteristics of studies included in the network meta-analysis

Study	Size	Follow-up (month)	Disease Stage	Age	Male (%)	Radiotherapy		Chemotherapy	
						Type	Dose/Gy	Intervention 1	Intervention 2
Al-Sarraf, 1998	193	30	AJCC III–IV,WHO I-III	50	67.0	RT	70	C (cisplatin) + A (fluorouracil, cisplatin)	Control
Cao, 2015	180	58.97	AJCC II–III	47	73.0	IMRT	70	C (cisplatin)	Control
Chan, 1995	82	28.5	WHO III	44	92.0	RT	58-66	N (fluorouracil, cisplatin)	Control
Chan, 2005	350	66	WHO I-III,UICC II-IV	45	80.0	RT	66	C (cisplatin)	Control
Chen, 2008	316	29	AJCC III–IVb	46	73.4	RT	70	C (cisplatin) + A (fluorouracil, cisplatin)	Control
Chen, 2011	220	60	AJCC II–III,WHO II-III	42	70.7	RT	68-70	C (cisplatin)	Control
Chen, 2012	508	37.8	WHO III-IVb	44	77.0	RT	66	C (cisplatin) + A (fluorouracil, cisplatin)	C (cisplatin)
Chi, 2002	157	49.5	WHO I-III	46	77.9	RT	70.2	A (leucovorin, fluorouracil, cisplatin)	Control
Chua, 1998	334	30	AJCC I–IV,M0	47	75.0	RT	71	N (epirubicin, cisplatin)	Control
Cvitkovic, 1996	339	49	WHO I-III,M0	42	75.0	RT	65-70	N (bleomycin, epirubicin, cisplatin)	Control
Ding, 2011	56	3	TNM II-IV	48	60.7	RT	70	C (cisplatin) + A (fluorouracil, cisplatin)	C (cisplatin)
Fountzilas, 2012	141	55	WHO I-III	49	71.0	RT	70	N (epirubicin, cisplatin, paclitaxel) + C (cisplatin)	C (cisplatin)
Ge, 2009	52	-	TNM II-III	54	76.9	RT	70	C (CMNa)	Control
Guan, 2016	69	35	AJCC I–IV,WHO II-III	48	85.7	IMRT	60	C (cisplatin)	Control
Hareyama, 2002	80	49	WHO I-III	50	75.0	RT	66-68	N (fluorouracil, cisplatin)	Control
Huang, 2012	200	-	WHO II-III	44	56.0	RT	66-78	N (fluorouracil, carboplatin) + C (carboplatin)	C (carboplatin)
Huang, 2015	408	133.3	UICC II-IV	45	77.6	RT	66-78	N (floxuridine, carboplatin) + C (carboplatin)	N (floxuridine, carboplatin)
Hui, 2009	65	-	UICC III-IV	50	61.8	RT	78.4	N (docetaxel, cisplatin) + C (cisplatin)	C (cisplatin)
Kong, 2015	200	-	WHO III-IV	50	63.0	RT	66-75	C (fluorouracil)	Control
Kwong, 2004*	219	37	AJCC II–IV,WHO I-III	45	69.1	RT	66	C (uracil, tegafur)	Control
								A (fluorouracil, cisplatin, vincristine, bleomycin, methotrexate)	C (uracil, tegafur) + A (fluorouracil, cisplatin , vincristine, bleomycin, methotrexate)
Lai, 2007	95	-	TNM I-IV	51	76.6	RT	70-80	C (CMNa)	Control
Lee, 2010	348	60	WHO III-Ivb	46	72.0	RT	68	C (cisplatin) + A (fluorouracil, cisplatin)	Control
Lee, 2011	441	73.2	WHO III-IVb	46	74.0	RT	66	C (cisplatin) + A (fluorouracil, cisplatin)	Control
Liang, 2008	72	-	TNM I-IV		62.2	RT	60-70	C (CMNa)	Control
Liao, 2008	48	-	TNM II-IV	51	58.3	RT	68-74	C (CMNa)	Control
Lin, 2003	284	65	WHO I-III	45	71.6	RT	70-74	C (fluorouracil, cisplatin)	Control
Liu, 2006	211	52	TNM I-IV	46	88.5	RT	68-70	C (CMNa)	Control
Liu, 2010	44	-	TNM III-IVa	51	72.7	RT	72-74	C (CMNa)	Control
Ma, 2001	456	62	WHO I-III	46	69.0	RT	68-72	N (bleomycin, fluorouracil, cisplatin)	Control
Ma, 2009	98	24	TNM III-Iva	48	77.6	RT	70	N (fluorouracil, cisplatin, paclitaxel) + C (fluorouracil, cisplatin)	C (fluorouracil, cisplatin)
Rossi, 1988	229	-	T1-4,N0-3	49	70.0	RT	60-70	A (vincristine, cyclophosphamide, adriamycin)	Control
Ruste, 2011	30	-	WHO III-IVb	36	62.5	RT	70	C (cisplatin) + A (fluorouracil, cisplatin)	N (fluorouracil, cisplatin) + C (cisplatin)
Tan, 2008	60	-	TNM I-Iva	51	50.0	RT	68-70	C (CMNa)	Control
Tan, 2015	172	40.8	WHO II-III	49	82.6	IMRT	70	N (paclitaxel, gemcitabine) + C (cisplatin)	C (cisplatin)
Wang, 2010	66	-	TNM III	45		RT	70-74	C (CMNa)	Control
Wang, 2014	695	66.4	WHO I-II	45	77.7	IMRT	67-76	C (cisplatin)	Control
Wee, 2015**	83	49.4	WHO I-Iib	49	68.7	IMRT	67.5	C (cisplatin)	C (cisplatin) + A (fluorouracil, cisplatin)
								N (docetaxel, fluorouracil, cisplatin or docetaxel, cisplatin or fluorouracil, cisplatin) + C (cisplatin)	-
Wen, 2014	60	-	AJCC III-Ivb	46	57.0	RT	60-66	C (docetaxel)	Control
Wu, 2006	40	-	TNM III-IV	56	75.0	RT	70-74	C (CMNa)	Control
Wu, 2013	115	114	WHO II-III			RT	70-74	C (oxaliplatin)	Control
Wu, 2014	35	31.9	UICC III-Ivb,WHO II-III	36	72.2	RT	70	C (H-R3)	Control
Xu, 2014	338	60	AJCC III-Ivb	49	74.1	RT	70-76	N (fluorouracil, cisplatin) + A (fluorouracil, cisplatin)	C (fluorouracil, cisplatin) + A (fluorouracil, cisplatin)
Xu, 2015	86	37.4	UICC II-IV	51	72.1	IMRT	66	C (cisplatin)	Control
Yang, 2007	60	-	T1-4N0-3M0	41	66.7	RT	60-70	C (CMNa)	Control
Yang, 2012	60	3	TNM II-IV	63	73.3	RT	72	C (CMNa)	Control
Yi, 2014	333	-	WHO III-IV	47	73.9	IMRT	70-74	C (cisplatin)	Control
Zeng, 2014	234	22	WHO II-III	48	86.0	RT	70	C (cisplatin)	Control
Zhang, 2005	115	24	WHO II-III,AJCC III–IV	46	67.8	RT	70-74	C (oxaliplatin)	Control
Zhang, 2008	100	-	TNM III-IV			RT	68-70	C (CMNa)	Control
Zhang, 2008	45	-	TNM III-IV	41	80.0	RT	70-74	C (CMNa)	Control
Zhang, 2015	799	55.27	WHO I-III	46	73.0	IMRT	60	N (docetaxel, paclitaxel, cisplatin or docetaxel, paclitaxel, cisplatin, fluorouracil) + C (cisplatin)	C (cisplatin)
Zhou, 2011	60	-	T2N2M0	46	80.0	RT	70-74	C (CMNa)	Control

Kwong, 2004*, four arms study; Wee, 2015**, three arms study; Abbreviations: AJCC, American Joint Committee on Cancer; WHO, World Health Organization; UICC, International Union Against Cancer; TNM, T, Tumor, N, regional lymph node, M, metastasis; RT, radiotherapy; IMRT, intensity-modulated radiotherapy; A, Adjuvant chemotherapy; C, Concurrent chemotherapy; N, Neoadjuvant chemotherapy; CMNa, sodium glycididazole.

### Result of conventional MA

Direct comparisons from conventional MA were shown in Table 2. NPC patients with C were associated with significantly decreased HR and increased probability of CR compared with the control group. The above trend was also presented in survival benefit between patients with C+A and those in the control group. Besides, patients with N+C exhibited an average increase of 9% in the 3-year OS (HR = 1.09, 95% CI = 1.01-1.16) in relation to those with C+A

**Table 2:** Meta-analysis results for pair-wise comparisons

Intervention 1	Intervention 2	5 years OS	3 years OS	1 years OS	CR
**A**	**Control**	1.22 (0.85, 1.75)	1.20 (0.81, 1.80)	1.16 (0.59, 2.28)	1.11 (0.86, 1.43)
**C**	**Control**	0.68 (0.52, 0.90)	0.66 (0.48, 0.90)	0.32 (0.15, 0.67)	1.16 (1.06, 1.28)
**C+A**	**Control**	0.65 (0.53, 0.80)	0.62 (0.47, 0.81)	0.46 (0.26, 0.81)	1.23 (0.81, 1.88)
**N**	**Control**	0.84 (0.69, 1.02)	0.86 (0.69, 1.06)	0.96 (0.63, 1.48)	1.04 (0.89, 1.21)
**C+A**	**A**	0.74 (0.36, 1.55)	0.69 (0.26, 1.84)	1.47 (0.03, 80.96)	-
**C+A**	**C**	0.80 (0.50, 1.29)	0.76 (0.40, 1.45)	1.10 (0.23, 5.20)	1.09 (0.72, 1.66)
**N+A**	**C+A**	0.84 (0.53, 1.34)	1.06 (0.27, 4.08)	-	-
**N+C**	**C**	0.94 (0.74, 1.21)	0.82 (0.43, 1.56)	0.59 (0.14, 2.53)	1.04 (0.83, 1.30)
**N+C**	**C+A**	-	1.09 (1.01, 1.16)	0.89 (0.38, 2.05)	-
**N+C**	**N**	1.04 (0.63, 1.71)	1.10 (0.54, 2.21)	0.82 (0.15, 4.47)	-

Abbreviation: A, Adjuvant chemotherapy; C, Concurrent chemotherapy; N, Neoadjuvant chemotherapy

### Result of NMA

Several trends were revealed by mixed-treatment comparisons, as recorded in Table 3 and shown graphically in Figure S1 and Figure S2. In the outcomes of 5-year OS, five therapies were associated with a significantly reduced HR compared with the control group (C: HR = 0.70, 95% CI: 0.59-0.85; C+A: HR = 0.64, 95% CrI: 0.52-0.79; N+C: HR = 0.74, 95% CrI: 0.57-0.96; N: HR = 0.80, 95% CI: 0.65-0.98; N+A: HR = 0.54, 95% CrI: 0.31-0.93). C, C+A and N+A also presented a decreased HR compared with A. With respect to 3-year, the result was similar to that during the five year period versus the control group. Similarity occurred in comparisons with A. Considering 1-year OS, significant result was obtained in the primary comparisons with control group and A, along with wider interval distributions. Additional significant result was achieved when we compared C, C+A and N+C with N (C: HR = 0.44, 95% CrI: 0.21-0.90; C+A: HR = 0.51, 95% CrI: 0.27-0.96; N+C: HR = 0.44, 95% CrI: 0.21-0.94), which may indicate the difference in the short-term performance. According to the result, we found that there was consistency among 1-year, 3-year and 5-year OS status. Furthermore, NPC patients with C, C+A, N+C and N appeared to have significantly higher possibility of CR compared with the control group.

**Table 3:** Network meta-analysis results for long-term and short-term prognoses

	3 years OS						
**5 years OS**	**Control**	1.16 (0.79, 1.70)	**0.70 (0.55, 0.90)**	**0.64 (0.52, 0.80)**	**0.80 (0.65, 0.99)**	0.68 (0.17, 2.70)	**0.72 (0.56, 0.91)**
	1.13 (0.79, 1.60)	**A**	**0.61 (0.39, 0.95)**	**0.56 (0.36, 0.86)**	0.69 (0.45, 1.07)	0.59 (0.14, 2.45)	**0.62 (0.40, 0.96)**
	**0.70 (0.59, 0.85)**	**0.62 (0.42, 0.93)**	C	0.92 (0.70, 1.20)	1.14 (0.84, 1.55)	0.97 (0.24, 3.88)	1.02 (0.77, 1.34)
	**0.64 (0.52, 0.79)**	**0.57 (0.39, 0.83)**	0.91 (0.70, 1.18)	**C+A**	1.24 (0.95, 1.63)	1.06 (0.27, 4.12)	1.11 (0.95, 1.31)
	**0.80 (0.65, 0.98)**	0.71 (0.47, 1.06)	1.13 (0.88, 1.45)	1.25 (0.93, 1.67)	**N**	0.85 (0.21, 3.41)	0.89 (0.68, 1.17)
	**0.54 (0.31, 0.93)**	**0.48 (0.25, 0.90)**	0.77 (0.44, 1.35)	0.84 (0.51, 1.39)	0.68 (0.38, 1.21)	**N+A**	1.05 (0.27, 4.15)
	**0.74 (0.57, 0.96)**	0.65 (0.42, 1.01)	1.05 (0.82, 1.33)	1.15 (0.83, 1.60)	0.92 (0.71, 1.20)	1.36 (0.75, 2.48)	**N+C**
	**CR**						

**1 year OS**	**Control**	1.73 (0.82, 3.78)	**3.06 (2.25, 4.28)**	**2.46 (1.16, 6.40)**	**2.89 (1.09, 8.98)**	-	**4.35 (1.89, 11.86)**
	1.12 (0.57, 2.18)	A	1.76 (0.77, 3.97)	1.41 (0.50, 4.86)	1.68 (0.47, 6.66)	-	2.51 (0.83, 8.63)
	**0.40 (0.21, 0.73)**	**0.35 (0.14, 0.88)**	**C**	0.81 (0.36, 2.04)	0.95 (0.34, 3.01)	-	1.43 (0.64, 3.52)
	**0.46 (0.28, 0.76)**	**0.42 (0.18, 0.95)**	1.17 (0.59, 2.34)	**C+A**	1.20 (0.30, 4.28)	-	1.79 (0.52, 5.70)
	0.91 (0.60, 1.37)	0.81 (0.37, 1.78)	**2.29 (1.11, 4.72)**	**1.95 (1.04, 3.65)**	**N**	-	1.51 (0.36, 6.03)
	-	-	-	-	-	**N+A**	
	**0.40 (0.20, 0.80)**	**0.36 (0.14, 0.93)**	1.01 (0.48, 2.11)	0.86 (0.45, 1.66)	**0.44 (0.21, 0.94)**	-	**N+C**

Abbreviation: A, Adjuvant chemotherapy; C, Concurrent chemotherapy; N, Neoadjuvant chemotherapy

5 years overall survival, 3 years overall survival, 1 year overall survival, represented by hazard ratio (HR) and 95% credible interval (CrI), and complete response represented by odds ratio (OR) and 95% credible interval (CrI). In lower half of the table, row treatments are compared against column treatments, whereas in the upper half, column treatments are compared against row treatments.

### Ranking of SUCRA

**Figure 3:**
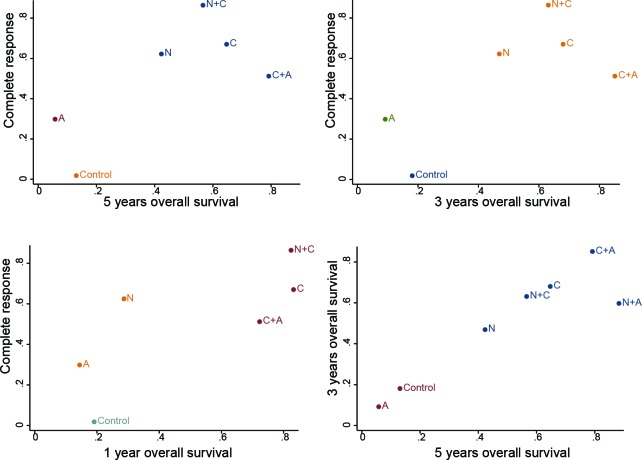
Clustered ranking plot of the network The plot is based on cluster analysis of surface under the cumulative ranking curves (SUCRA) values. Each plot shows SUCRA values for two outcomes. Each color represents a group of treatments that belong to the same cluster. Treatments lying in the upper right corner are more effective and safe than the other treatments.

Firstly, the SUCRA values revealing the rank of abovementioned therapies in different outcomes were recorded in Table 4. Overall, C together with the combined approaches of C+A and N+C exhibited to be the most competitive therapies with respect to prognostic outcomes and complete response according to the SUCRA values. Another noteworthy therapy was N+A, it was the most efficacy combination in 5y-OS outcomes. Then, the result was well displayed by the cluster analysis in Figure 3, in which the included therapies were categorized into different groups based on their SUCRA values. Cluster analysis suggested that the above three chemoradiotherapy appeared to be divided into the most efficacious group located in the upper right corner of the cluster plots. On top of that, the cluster plots showed that the results of included outcomes were substantially similar in this NMA.

**Table 4:** Surface under the cumulative ranking curve (SUCRA) values of each intervention

Interventions	5 years OS	3 years OS	1 years OS	CR
**Control**	0.131	0.181	0.191	0.018
**A**	0.058	0.092	0.144	0.298
**C**	0.647	0.680	0.832	0.670
**C+A**	0.792	0.851	0.723	0.512
**N**	0.423	0.469	0.285	0.622
**N+A**	0.883	0.597	-	-
**N+C**	0.566	0.631	0.824	0.864

Abbreviation: A, Adjuvant chemotherapy; C, Concurrent chemotherapy; N, Neoadjuvant chemotherapy

### Consistency between direct and indirect evidence

**Figure 4:**
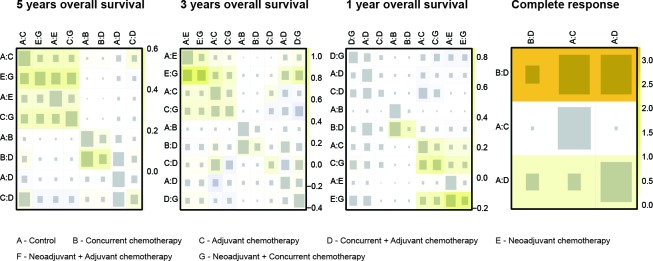
Net heat plot Warm color in the net heat plot indicates that significant inconsistency may arise from a specific design or comparison and this trend is illustrated by the intensity of the color.

Since the consistency model was introduced in the NMA, this assumption was supposed to be evaluated in this NMA. As suggested by the net heat plot in Figure 4, no significant inconsistency appeared in the comparison with respect to the survival outcomes. However, substantial inconsistency was observed from the comparison between C and C+A under the endpoint of CR (*P* = 0.041), as was shown in the node splitting plot (Figure S3).

## DISCUSSION

In this study, a comprehensive and quantitative comparison among the existed chemotherapies given as the integral part of radiotherapy in patients with NPC was successfully conducted. This evaluation presented both direct and indirect evidence through pairwise meta-analysis and mixed meta-analysis. The statistical differences presented in the results would lead us to give an appropriate estimate and find out the optimal clinical choices.

As the SUCRA results revealed, therapeutic strategies based on concurrent chemoradiotherapy, including concurrent chemoradiotherapy alone or combined with adjuvant or neoadjuvant chemotherapy, were recommended as the first-line therapies, when the characteristics of individuals are not clarified. It was reported that platinum-based concurrent chemoradiotherapy had been accepted by the National Comprehensive Cancer Network as the standard recommendation for locally advanced NPC since the Intergroup 0099 study was published in 1998 [7]. And its superiority in clinical performance over traditional radiotherapy had been demonstrated in subsequent RCT studies [8] and MAs [9, 10]. It was reported that tissue fibrosis and vascular changes of tumor in locally recurrent NPC attributed to its poor sensitivity to radiotherapy [11]. However, chemotherapy was found to be a highly responded therapy instead [12]. Thus concurrent chemotherapy provided a reinforced efficacy by increasing the sensitivity of NPC toward radiotherapy. When comparing the add-on chemotherapies based on concurrent chemoradiotherapy (C+A and N+C), no head-to-head comparison was conducted according to the retrieve results. Besides, no statistically significant result had been pooled, with merely marginal differences. Additionally, the survival benefit in the documented records was as ambiguous as the NMA result, meaning that the addition of adjuvant or neoadjuvant chemotherapy had not been significantly translated to the improvement in overall survival benefit and complete response rate.

Among those chemotherapies binding with conventional radiotherapy, the number of included publications was limited, which meant further evidence was still required to give a more accurate evaluation. Neoadjuvant chemotherapy, followed by radiotherapy alone with adjuvant chemotherapy, is given an appreciable preference, especially in the outcomes of 5-year overall survival rate, although recorded by merely one publication, which is documented as a long-term, updated result reported by *Xu et al* [13]. Undoubtedly, the relevant ranking was lack of solid credibility. However, according to this document, this kind of chemotherapy added to traditional radiotherapy was comparable with concurrent plus adjuvant chemoradiotherapy in the aspect of overall survival benefits, and was considered as a potential alternative to the latter. As a result, this potentially preferable therapy could be a research focus of further RCT studies. The separated neoadjuvant chemotherapy followed by radiotherapy was documented by 5 publications, among which the latest one was updated in 2002 by *Hareyama et al* [14]. It had a middle position in the SUCRA ranking score and presented a moderate performance in the including outcomes. However, its limitation was reported as a low proportion of patient response [15], failure to achieve the primary goal of eradicating distant micro-metastases because of non-sufficient dosage [16] or a failed translation from the reduction in local relapses into an overall survival advantage due to local or regional recurrence [16]. As a result, almost no further researches were reported and neoadjuvant chemotherapy became an integral part of concurrent chemoradiotherapy. Adjuvant chemotherapy plus radiotherapy exhibits to be not advantageous in the case of improving overall survival and tent to result in more complete response than the control group of radiotherapy. The included publication pooled no significant improvement, just consistent with our NMA. It was criticized for the regimen was thought to be suboptimal [17] or dose-intensity was reduced [17]. Despite the low ranking in our NMA result, adjuvant chemotherapy was reported to be efficacious in decreasing the chance of systemic relapse [18]. Consequently, a more reasonable estimate would be given if outcomes were taken into consideration.

Radiotherapy alone seemed to be the last choice for patients with NPC. Although it was considered useful in the early-stage NPC, the low cure rate made it unsatisfying [19, 20], which was coincident with the result that we had demonstrated in NMA. Especially in those with locally recurrent NPC, re-irradiation is associated with severe complications at high doses, which could even be the primary cause of death [21]. The confirmed therapeutic advantage of concurrent chemoradiotherapy over radiotherapy was the result of the fact that most included trials were comparing with the conventional technique. However, there had been development in this traditional therapy. Intensity-modulated radiotherapy (IMRT), a modern radiotherapy technique, had been applied in more studies, especially in the recent trials [22–24]. In this case, the tumor volumes were delineated more accurately, so better dose distribution could be adopted [22]. Thus, significant improvement has been diluted when patients were given the additional chemotherapy. Besides, three-dimensional conformal radiation therapy (3D-CRT) was another developed technique, providing improved calculation, shielding, and the classic field arrangement compared with the traditional 2D technique [25]. The primary purpose of chemotherapy is to increase the disease control locally and distantly [26, 27]. And its advantageous clinical performance had been proved in a large quantity of trials. Nevertheless, the tendency had emerged that advanced radiotherapy technology could be alternative to concurrent chemoradiotherapy.

Though successfully conducted, a number of limitations still existed in this NMA. First, the detailed information that was directly related to survival rate, such as distant metastasis, and the toxicity of chemotherapy with the corresponding adverse responses was not included. Though most adverse responses were manageable and uncomplicated, and not associated with death, chemotherapy was still poorly tolerable. Moreover, undoubtedly the addition of chemotherapy was responsible for some severe events. For example, the increase of hematologic events in patient with neoadjuvant or adjuvant chemotherapy had been reported [23]. Second, as we had mentioned above, specific comparison was limited due to the lack of available head-to-head trials, especially among those chemotherapies based on traditional radiotherapy. It was insufficient to pool a clear result and give a critical conclusion. Finally, we did not use the detailed data of individual patients. In fact, the included patients are belonging to different stages of NPC sufferers. And they were characterized by different symptoms, so researchers were tent to divide them into subgroups. It had been proposed that radiotherapy alone can be sufficient treatment for early-stage NPC patients. Combined concurrent chemoradiotherapy with adjuvant chemotherapy was recommended for those at intermediate risk stage. Aggressive neoadjuvant chemotherapy, followed by CCRT and adjuvant chemotherapy may be the choice for high-risk patients [28].

In conclusion, in view of survival rate and complete response, concurrent chemoradiotherapy with adjuvant chemotherapy (C+A), concurrent chemoradiotherapy with neoadjuvant chemotherapy (N+C)and concurrent chemoradiotherapy (C) itself was considered as the first-line treatment according to the NMA result. Even so, it was worth noting that the advanced modern radiotherapy technique had the potential to be an alternative. Cautious and approaches based on evidence should be maintained. Guidance from our NMA was recommended to be integral with individual characteristics.

## MATERIALS AND METHODS

### Search strategy and selection criteria

Literature search was conducted in electronic databases by two independent reviewers. Multiple resources were searched accordingly for the purpose of preventing selection bias: China National Knowledge Internet (CNKI), PubMed and Embase. Literature search was not restricted to any language or type of publication. The following key terms accompanied with their entry terms were used to build a searching query: “nasopharyngeal neoplasms”, “radiotherapy”, “chemotherapy”, “chemoradiotherapy” and etc. The searching results were updated in September 2016.

Studies were included if they were randomized controlled trials (RCT) which combined at least one chemotherapy regimen with radiotherapy. Besides that, patients in the included studies [1–11, 13, 14, 17, 24, 29–65] were diagnosed with NPC (stage I to IV) according to the criteria set by the American Joint Committee on Cancer (AJCC), the World Health Organization (WHO), the International Union against Cancer (IUAC) and the tumor node metastasis (TNM) staging system.

### Data extraction

The process of data extraction was accomplished by two independent reviewers. The following study characteristics were included for each research: (1) the basic information of the research, including the first author, published year, the size of samples and the follow-up duration; (2) the patients characteristics, including tumor stage, age and sex; (3) the regimens, including the type and dose of radiotherapy and the interventions for comparison; (4) outcomes, including 1-year, 3-years and 5-years overall survival (OS) and complete response (CR). If the same study had been published for more than once, the one with longer following-up duration would be preferred. risk of bias was also evaluated by using the famous Cochrane risk of bias assessment tool [54]. Each study was classified as having high, low or unclear risk of bias.

### Statistical analysis

Since not only the short-term efficacy of chemoradiotherapies but also their long-term efficacy in NPC patients was concerned in this NMA, the 3-year and 5-year OS were selected as major outcomes whereas the 1-year and CR were selected as secondary outcomes. We used the hazard ratio (HR) and its 95% confidence intervals (CIs) to measure the relative efficacy between two comparators when survival data were synthesized from individual studies. A significantly increased HR (HR > 1) suggested that one therapy may be less efficacious than another and vice versa. Besides, the statistics of odds ratio (OR) and its 95% (CIs) were also computed when CR data were synthesized from individual studies. The random-effects model was introduced for pair-wise meta-analysis, which generated summary statistics for every direct comparison. We used *I****2*** statistics to evaluate the between-study heterogeneity, in which *I****2*** > 50% was considered high heterogeneity. Then R 3.2.3 and STATA 13.0 were adopted to conduct NMA. Summary statistics and their 95% credible intervals (CrIs) were computed by the approach of NMA. Furthermore, the cumulative ranking probability of each chemoradiotherapy was computed so that chemoradiotherapy can be ranked with respect to each outcome. Additionally, the net heat plot was created by the software to evaluate the consistency between direct and indirect comparison. Warm color in the net heat plot indicates that significant inconsistency may arise from a specific design or comparison and this trend is illustrated by the intensity of the color. Besides, the node splitting method was adopted to test the presence of significant inconsistency for each comparison and *P*-value < 0.05 concludes the significance of inconsistency. Finally, chemoradiotherapies were categorized into different groups by using the approach of cluster analysis.

## SUPPLEMENTARY MATERIALS FIGURES AND TABLES



## Abbreviations

OS: overall survival,
CR: complete response
NPC: nasopharyngeal carcinoma
NMA: network meta-analysis
HR: hazard ratio
C: concurrent chemoradiotherapy
A: adjuvant chemotherapy
N: neoadjuvant chemotherapy
C+A: concurrent chemoradiotherapy with adjuvant chemotherapy
C+N: concurrent chemoradiotherapy with neoadjuvant chemotherapy
N+A: neoadjuvant chemotherapy with adjuvant chemotherapy
CIs: confidence intervals
OR: odds ratio
RT: radiotherapy
RCTs: randomized clinical trials
CNKI: China National Knowledge Internet
AJCC: American Joint Committee on Cancer
WHO: World Health Organization
IUAC: International Union against Cancer
TNM: tumor node metastasis.
