# Supplementary material for: Chemoradiotherapy enhanced the efficacy of radiotherapy in nasopharyngeal carcinoma patients: a network meta-analysis
Source: Oncotarget. 2017 Mar 17;8(24):39782–94. doi: 10.18632/oncotarget.16349 (PMC5503653; doi:10.18632/oncotarget.16349)
Supplement: Supplementary file 1 [file oncotarget-08-39782-s001.pdf]

Chemoradiotherapy enhanced the efficacy of radiotherapy in nasopharyngeal carcinoma patients: a network meta-analysis

Supplementary Material

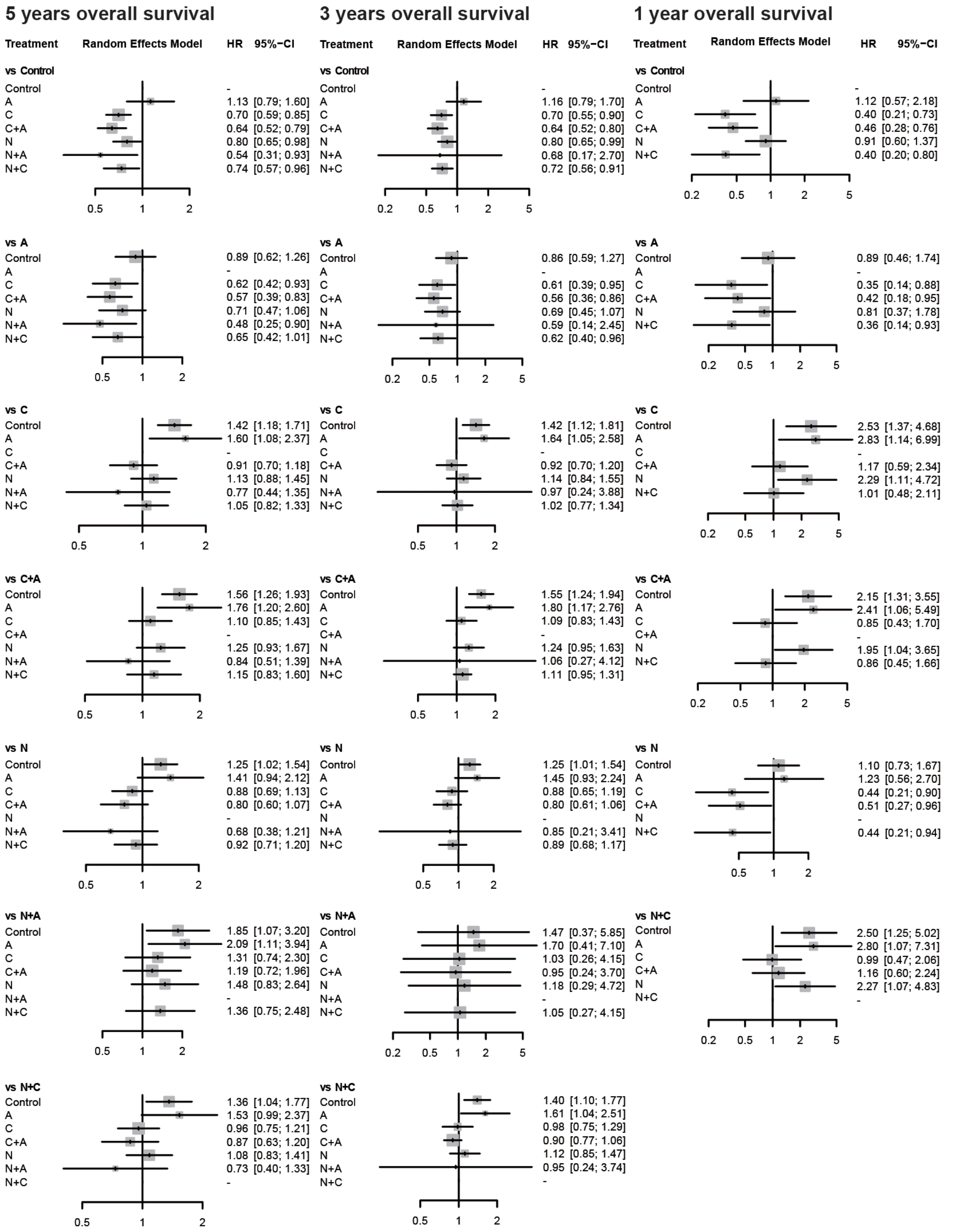

Figure S1: Forest plot for 5-year, 3-year and 1-year overall survival.

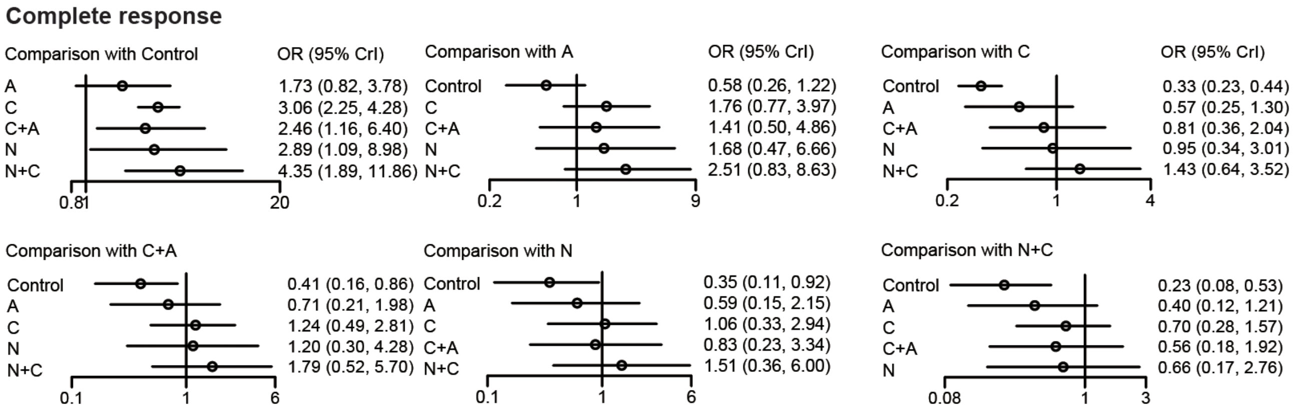

Figure S2: Forest plot for complete response.

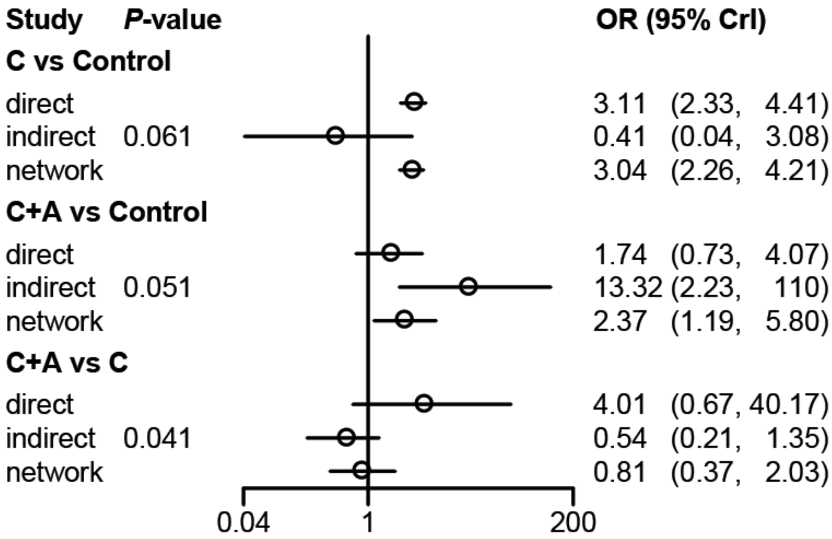

Figure S3: Node splitting results for complete response.
